# Supplementary material for: Downregulation of Let-7 miRNA promotes Tc17 differentiation and emphysema via de-repression of RORγt
Source: bioRxiv. 2024 Mar 4:2023.10.12.562059. Preprint. [Version 3] doi: 10.1101/2023.10.12.562059 (PMC10614797; doi:10.1101/2023.10.12.562059)
Supplement: Supplement 1 [file media-1.pdf]

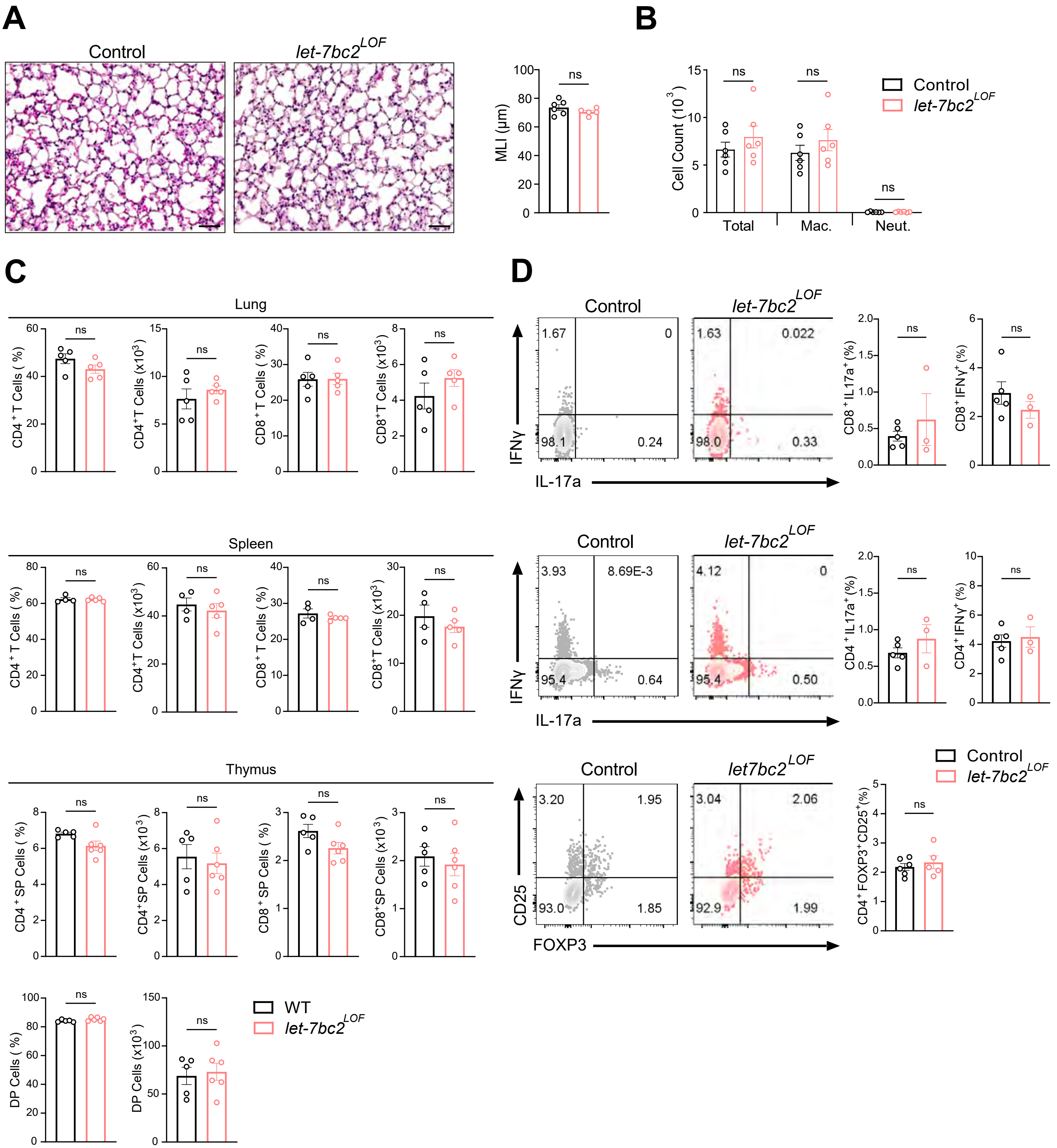

Figure 2-figure supplement 1. T cell-specific deletion of the *let-7bc2*-cluster does not promote lung inflammation or pathology with moderate aging. (A) Representative H&E-stained lung sections from Control and *let-7bc2*^{LOF} naive mice aged to one year (x20 magnification; scale bars, 50μm) with MLI measurement of lung morphometry (n=5-6 per group). (B) Total and differential cell count from bronchoalveolar lavage (BAL) fluid from control and *let-7bc2*^{LOF} naive mice (n=6 per group; Mac. = macrophages, Neut. = neutrophils). (C) Flow cytometric analysis of CD4^{+}, CD8^{+}, or DP T cells from the lungs, spleen, and thymus of control and *let-7bc2*^{LOF} mice at steady-state (n=4-6 per group). (D) Immunophenotyping of Tc17, Tc1, Th17, Th1, and Tregs from lungs of naïve control and *let-7bc2*^{LOF} mice (n=3-5 per group). Data are representative of three independent experiments and displayed as mean±SEM using student's t-test.
