# Supplementary material for: Downregulation of Let-7 miRNA promotes Tc17 differentiation and emphysema via de-repression of RORγt
Source: bioRxiv. 2024 Mar 4:2023.10.12.562059. Preprint. [Version 3] doi: 10.1101/2023.10.12.562059 (PMC10614797; doi:10.1101/2023.10.12.562059)
Supplement: Supplement 4 [file media-4.pdf]

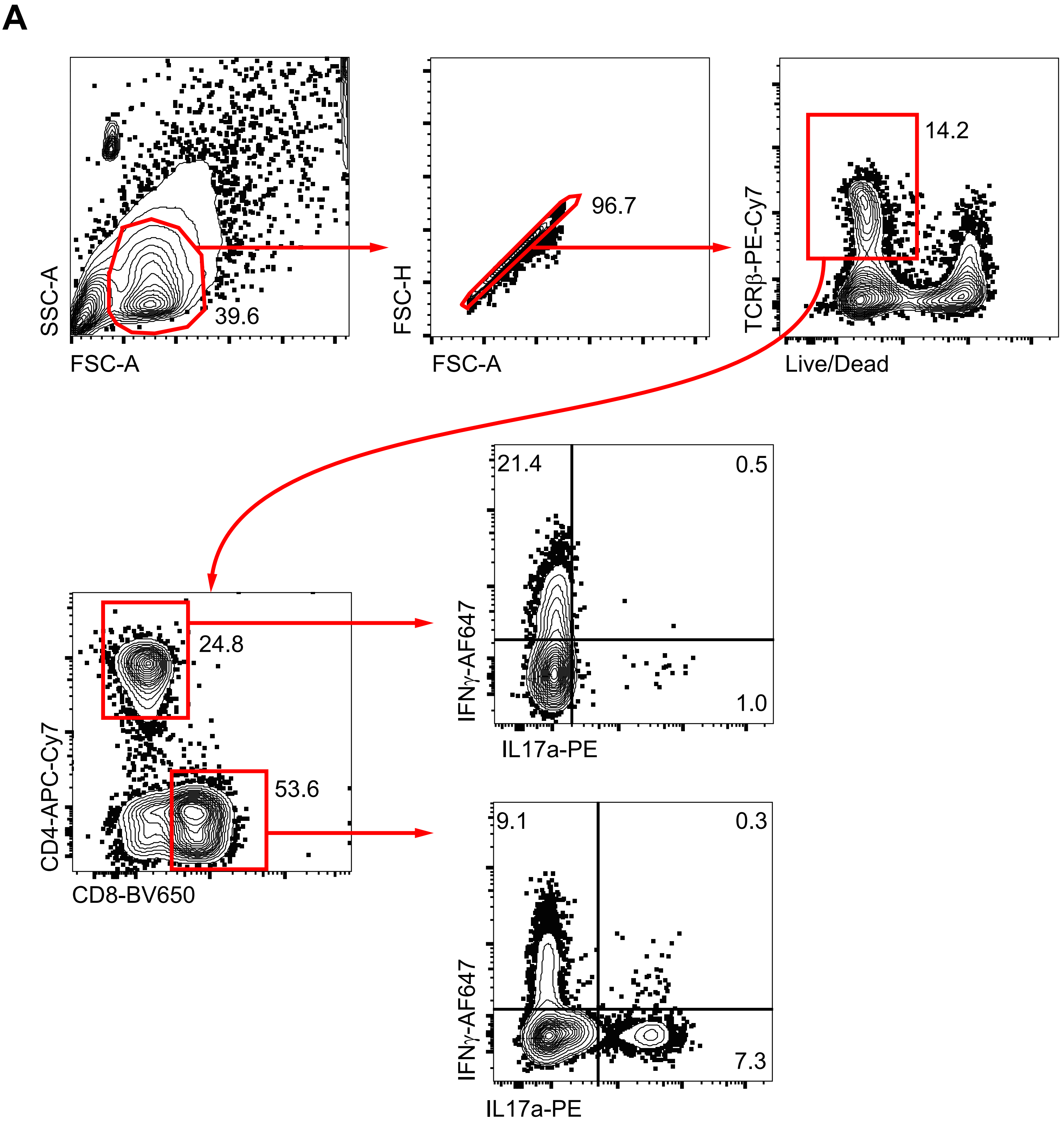

Supplementary Figure 3. (A) Representative flow gating strategy for the identification of Th1/Th17 and Tc1/Tc17 T cell subsets in mouse lungs of wild-type control mouse.
