# Supplementary material for: Downregulation of Let-7 miRNA promotes Tc17 differentiation and emphysema via de-repression of RORγt
Source: bioRxiv. 2024 Mar 4:2023.10.12.562059. Preprint. [Version 3] doi: 10.1101/2023.10.12.562059 (PMC10614797; doi:10.1101/2023.10.12.562059)
Supplement: Supplement 5 [file media-5.pdf]

Supplementary Table 1: Genotyping Primers and Duplexes for Luciferase Assay

Genotyping Primers

**Name**	**Sequence**	**Description**
BC-lox2-F	5'-GGACATGAGATCGCCAACCA-3'	*let-7bc2*-cluster floxed allele<br>Forward primer for genotyping
BC-lox2-R	5'-TGGAAGCCAGTACTGTGCTC-3'	*let-7bc2*-cluster floxed allele<br>Reverse primer for genotyping
AFD-lox2-F	5’-GTTTTCTGAGGTGTGGGAGGTA-3’	*let-7afd*-cluster floxed allele<br>Forward primer for genotyping
AFD-lox2-R	5’-AGTGGGATAGAAGGATCTCAGG-3’	*let-7afd*-cluster floxed allele<br>Reverse primer for genotyping

Dharmacon Duplexes

**Name**	**Sequence**
hsa-let-7b-5p	5'-UGAGGUAGUAGGUUGUGUGGUU-3'
Control (*cel-miR-67-3p*)	5'-UCACAACCUCCUAGAAAGAGUAGA-3'
