# Supplementary material for: Downregulation of Let-7 miRNA promotes Tc17 differentiation and emphysema via de-repression of RORγt
Source: bioRxiv. 2024 Mar 4:2023.10.12.562059. Preprint. [Version 3] doi: 10.1101/2023.10.12.562059 (PMC10614797; doi:10.1101/2023.10.12.562059)
Supplement: Supplement 6 [file media-6.pdf]

Supplementary Table 2. Demographics of subjects by emphysema severity

Emphysema Severity Score	= 0	= 1	= 2	= 3
Sex, n (%)				
Male	2 (100)	5 (100)	5 (100)	6 (85.7)
Female	0 (0)	0 (0)	0 (0)	1 (14.3)
Age	74.0 ± 4.2	66.0 ± 7.6	65.2 ± 4.6	67.1 ± 3.9
Race, n (%)				
Caucasian	2 (100)	5 (100)	4 (80)	5 (71.4)
African American	0 (0)	0 (0)	1 (20)	2 (28.6)
Current Smoker, n (%)				
Yes	0 (0)	2 (40)	3 (60)	4 (57.1)
No	2 (100)	3 (60)	2 (40)	3 (42.9)
FEV_1 %	57.0 ± 17.0	68.8 ± 12.3	74.2 ± 13.9	80.7 ± 9.5
FEV_1/FVC %	68.5 ± 12.0	66.6 ± 5.7	70.3 ± 9.4	60.3 ± 6.7

Mean ± standard deviation is shown unless otherwise stated.
